# Preoperative inspiratory muscle training preserved diaphragmatic excursion after esophagectomy: a randomized-controlled trial

**DOI:** 10.1007/s10388-025-01123-w

**Published:** 2025-04-03

**Authors:** Hiroki Mizusawa, Yuji Higashimoto, Osamu Shiraishi, Masashi Shiraishi, Ryuji Sugiya, Masaya Noguchi, Kengo Kanki, Tamotsu Kimura, Akira Ishikawa, Takushi Yasuda

**Affiliations:** 1 Faculty of Medicine, Department of Rehabilitation Medicine, Kindai University, 377-2, Ohno-higashi, Osakasayama, 589-5811 Japan; 2 Department of Public Health, Graduate School of Health Sciences, Kobe University, Kobe, Japan; 3 Department of Respiratory Medicine and Allergology, School of Medicine, Kindai University, Osaka, Japan; 4 Faculty of Medicine, Department of Surgery, Kindai University, Osaka, Japan

**Keywords:** Esophagectomy, Respiratory muscle training, Diaphragm, Ultrasonography, Maximal inspiratory pressure

## Abstract

**Background:**

Preoperative inspiratory muscle training (IMT) enhances diaphragmatic excursion (DE) in patients with esophageal cancer; however, the postoperative effects of IMT on DE have not yet been evaluated. This study aimed to investigate the effect of preoperative IMT on perioperative diaphragmatic function as measured by DE, inspiratory muscle strength, lung function, and exercise tolerance.

**Methods:**

This was a parallel, randomized-controlled trial. Patients with thoracic or abdominal esophageal cancer scheduled for esophagectomy were randomized into either the incentive spirometry (IS) or IMT group. Each intervention was performed during preoperative neoadjuvant chemotherapy (NAC). The primary outcome was the DE, and the secondary outcomes were maximal inspiratory pressure (MIP), lung function, and exercise tolerance, which were measured before and 1–3 months after esophagectomy.

**Results:**

Thirty-two patients were included in the analysis. The DE in the IMT group (*n* = 15) increased from baseline to pre-operation, and the rate of change was significantly greater than that in the IS group (*n* = 17). Although the DE and MIP decreased in both groups after esophagectomy, the decline in the DE after esophagectomy was significantly lower in the IMT group than that in the IS group (*p* < 0.05). Furthermore, significant differences in DE persisted at least until 3 months post-esophagectomy, whereas MIP did not differ significantly at any time point. Pulmonary function and exercise tolerance were not significantly different between the two groups.

**Conclusions:**

The IMT before esophagectomy enhanced diaphragmatic function, which was preserved for more than 3 months after esophagectomy.

**Supplementary Information:**

The online version contains supplementary material available at 10.1007/s10388-025-01123-w.

## Introduction

Esophageal cancer is the eighth most common cancer worldwide [1]. Esophagectomy plays a significant role in achieving locoregional control, and is the best option for local and advanced disease treatment for esophageal cancer. However, it is the most invasive type of thoracic-abdominal surgery [2, 3]. Therefore, esophagectomy has a high incidence of postoperative pulmonary complications (PPCs) [4]. Several systematic reviews have shown that inspiratory muscle training (IMT) in upper abdominal surgery has a positive effect on the prevention of PPCs [5, 6]. The main target muscle of IMT is the diaphragm, which is responsible for the majority of inspiration, and IMT provides an inspiratory load to the diaphragm by applying a controlled amount of inspiratory resistance. On the other hand, incentive spirometry (IS) has been used as a preoperative intervention for the prevention of PPCs after upper abdominal surgery, wherein deep breathing is performed with a device that provides visual feedback and is considered to maximize accuracy and motivation in breathing techniques [7]. In our previous study, we conducted a randomized-controlled trial in patients with esophageal cancer scheduled for esophagectomy, with two interventions in the preoperative period: IMT and IS. The results showed that preoperative IMT tended to lower the incidence of PPCs with enhanced diaphragmatic excursion (DE) [8].

In an observational study, vital capacity (VC) and forced expiratory volume in 1 s (FEV_1_) after open or non-open esophagectomy were lowest at 3 months postoperatively and remained lower than preoperative values until 12 months postoperatively [9]. Thus, diaphragmatic function and respiratory muscle strength may also decline over the first 3 months after esophagectomy, with poor recovery thereafter. Preoperative IMT intervention in upper abdominal surgery increased maximal inspiratory pressure (MIP) and VC, and were higher than those in the control group, even when measured on the seventh postoperative day [10]. However, no studies have followed and reported diaphragmatic function and respiratory muscle strength after the acute postoperative phase of patients with esophageal cancer who underwent preoperative IMT intervention. The IMT before esophagectomy may inhibit the postoperative decline in inspiratory muscle and pulmonary function. The purpose of this study was to follow up on the DE and respiratory muscle strength of patients who underwent esophagectomy in our randomized-controlled study of preoperative IMT and IS. In addition, we investigated whether these variables had a difference from baseline (before the intervention) to 3 months after esophagectomy between the two groups.

## Material and methods

### Patients

The inclusion criteria were thoracic and abdominal esophageal squamous cell carcinoma treated with neoadjuvant chemotherapy (NAC), followed by esophagectomy via right thoracotomy/thoracoscopy at the Kindai University Hospital, Osaka, Japan, from September 2020 to August 2023. The exclusion criteria were as follows: (1) patients aged > 80 years, (2) those who underwent two-phase surgery, (3) those who underwent esophagectomy as salvage treatment after chemoradiotherapy, (4) those with nasogastric feeding, (5) those who declined to participate in this study, and (6) those who did not provide an explanation for obtaining consent. NAC comprising docetaxel, cisplatin, 5-fluorouracil, and docetaxel (DCF), or 5-fluorouracil, docetaxel, and nedaplatin (UDON) was administered to patients with clinical stage II–III esophageal squamous cell carcinoma [11–13]. This study was approved by the Ethics Committee of Kindai University Faculty of Medicine (no. 31–252) and was conducted in accordance with the ethical standards of the 1964 Declaration of Helsinki and its subsequent amendments. All participants received sufficient information about the study, and written consent was obtained from all participants.

### Randomization

In this unblinded randomized study, patients were randomly assigned to either the IS or the IMT group using nondeterministic minimization method with two assignment factors: age and sex. A parallel design was used in this study. Eligible participants were randomly assigned to the IS (control) or IMT (intervention) group at a ratio of 1:1 by a person who was not involved in this study, using web-based randomization software (Mujinwari; Iruka System, Tokyo, Japan). The DE was measured by the same physical therapist, who was very skilled in measuring the DE and was blinded to which group the enrolled participants were assigned. The type of intervention was disclosed after enrolling all the data. The trial was registered with the University Hospital Medical Information Network of Japan on May 31, 2020 (ID:000042075).

### IS and IMT

#### IS group

The Coach2^®^ Incentive Spirometer 2500 ml (Smiths Medical ASD, Inc., USA, Online Resource 1a) was used to perform deep breathing exercises in the IS group. Coach2^®^ was visualized using a raised plate in a transparent cylinder during sustained inspiration. On a calibrated scale in the cylinder, the raised plate of the spirometer displayed the inspiratory volume. The participants were instructed, while holding the device in the mouth, to take deep breaths from maximal expiration to maximal inspiration at a constant inspiratory flow rate to raise the plate in the cylinder as much as possible. The MIP was measured every 2 weeks.

#### IMT group

The POWERbreathe^®^ Medic (POWERbreathe International Ltd., USA, Online Resource 1b) was used in the IMT group. Inspiratory resistance was set at 30%–50% MIP. The intervention in the first week was set at 30% MIP to allow participants to become familiar with the equipment and loading. Subsequently, it was then set at 50% MIP. Previous studies have reported that diaphragm muscle activity was greatest at 50% MIP in healthy participants. Moreover, the diaphragm muscle activity plateaued at higher loading pressures [14]. The MIP was measured every 2 weeks, and the IMT loading pressure was adjusted once every 2 weeks; hence, the IMT loading pressure was set to 50% MIP.

#### Commonalities between the IS and IMT groups

The minimum number of trials in the IS and MIP groups was at least twice per day, with 30 breaths per set, and were performed at least 4 days per week. An implementation status recording form was also distributed, and the number of trials performed each day was recorded. The intervention for each group was conducted in the preoperative period, and neither group performed any device-based respiratory training after esophagectomy. All measurements were performed the day before the initiation of neoadjuvant therapy (baseline T0) and a few days before esophagectomy (pre-op T1). At postoperative follow-up, all measurements were performed at 1 (post-op T2) and 3 months (post-op T3) after esophagectomy.

### Measurements

#### DE

The DE of the right hemidiaphragm was measured using the Xario 200^™^ (Toshiba, Tokyo, Japan) with a convex 3.5-MHz probe according to the technique described by Testa et al. [15]. The probe was placed longitudinally under the right costal arch to measure the DE of the right posterior one-third of the diaphragm. Furthermore, the participants remained in a standing position during the DE measurement (Online Resource 2a). The liver and kidneys on ultrasonography (US) displayed in B-mode were used as references (Online Resource 2b). The M-mode cursor was rotated and positioned along the axis of the diaphragmatic displacement on the stored image. The participants were instructed to take three deep breaths, and each breath was measured. This study used the largest amount of data available. The DE was measured between the maximal expiration and inspiration levels (displacement measurements; Online Resource 2c). The diaphragm mobility was reliably and reproducibly assessed using US [16].

#### Spirometry and MIP measurement

Pulmonary function was assessed using the CHESTAC-8800 (Chest, Tokyo, Japan). Spirometry was performed according to the 2019 American Thoracic Society recommendations for measuring forced VC, FEV_1_, and inspiratory capacity [17]. The respiratory muscle strength testers (IOP-01, Kobata Keiki Co., Osaka, Japan) were used to measure the MIP, which were repeated until the maximal inspiratory error of the three trials was < 10%, as per the European Respiratory Society statement [18]. The maximal value was used for the analysis. Based on the report by Hamada et al. [19], the MIP-predicted values (MIP in men: 45 − 0.74 × age + 0.27 × height (cm) + 0.6 × weight (kg) and MIP in women: − 1.5− 0.41 × age + 0.48 × height (cm) + 0.12 × weight (kg)) were used to calculate the ratio of the actual value to the predicted value (%pred. MIP).

#### Cardiopulmonary exercise testing

The cardiopulmonary exercise testing on a bicycle ergometer was conducted in accordance with the statements of the American Thoracic Society and the American College of Chest Physicians [20]. All patients underwent the ramp 20-W protocol (load increase of 20 W per 1 min or 2 W per 6 s). Exercise tolerance was assessed on the basis of peak oxygen consumption (peak VO_2_, mL/min/kg).

#### Evaluation of PPCs

PPCs were defined as atelectasis/difficulty excreting sputum (e.g., need for bronchoscopy), pneumonia, initial ventilatory support for > 48 h, and reintubation due to respiratory failure. Postoperative pneumonia was defined as new or progressive infiltration on chest radiography or CT scan and any of the following criteria: temperature of > 38.0 °C and white blood cell count of > 10,000/µL. Atelectasis is defined as lung opacification with mediastinal shift, hilum, or hemidiaphragm shift toward the affected area, with compensatory hyperinflation in adjacent non-atelectatic lung [21]. With these criteria, the diagnosis of PPCs was determined by the surgeon in all cases. Furthermore, all PPCs were graded using the Clavien–Dindo classification, and complications were defined as grade II or higher [22].

### Sample size

Unpublished preliminary and pilot data from healthy adults were used to calculate the sample size. The mean difference in the DE following the intervention change was 1.0 mm (standard deviation of 5.9) in the IS groups and 7.0 mm (standard deviation of 5.7) in the IMT group. We hypothesized that the incorporation of IMT would increase this change from 1.0 mm to 7.0 mm. The standard deviation of the change was assumed to be 6.0 mm in both groups. Under these conditions, and with the normal distribution of the DE, we hypothesized that the superiority of IMT over IS could be evaluated using a *t* test. Assuming a significance level of 5% (two-tailed) and power of 80%, the required number of cases was 32 (16 in each group); hence, the target number of patients was set to 40, considering dropouts. In our previous study, the primary endpoint was DE, and we set the sample size based on the intervention period from the initiation of NAC to before esophagectomy [8].

### Statistical analysis

Statistical analyses were performed using the Statistical Package for the Social Sciences software version 22 (IBM Corp., Armonk, NY, USA). All data were presented as mean and standard deviation or frequency and proportion (%). Unpaired *t* tests were used to compare patient characteristics and mean change from baseline between the two groups.

Repeated paired *t* tests were used for comparisons at baseline, T1, T2, and T3 time points within each of the two groups. Multiple comparisons within each group were performed for T1, T2, and T3 using the Bonferroni adjustment. Categorical variables were performed with Fisher's exact test or *χ*^2^ test. Statistical significance was set at *p* < 0.05.

## Results

### Characteristics of the participants

**Fig. 1:**
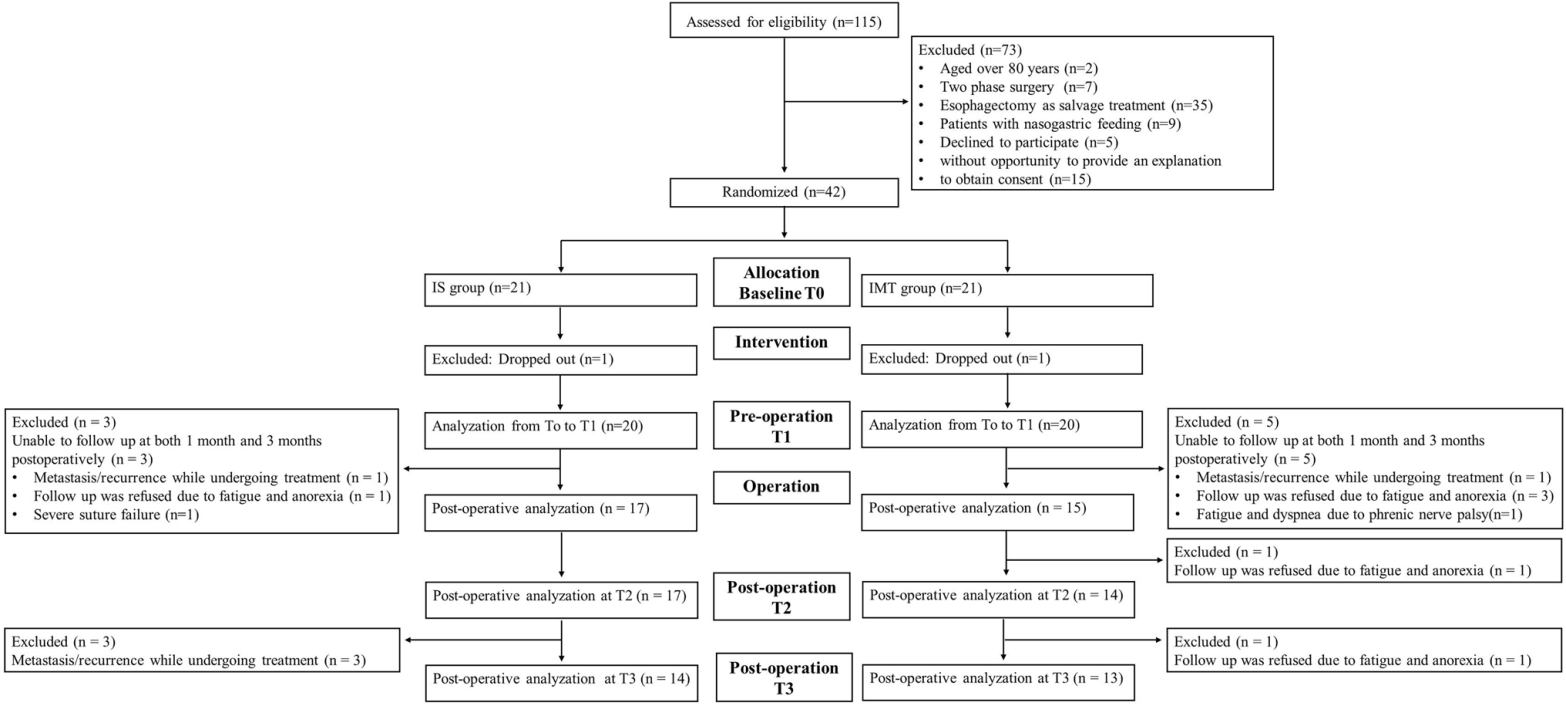
Flowchart of the patient selection process. Allocation baseline T0: day before the initiation of neoadjuvant therapy. Pre-operation T1: a few days before esophagectomy. Post-operation T2: 1 month after esophagectomy. Post-operation T3: 3 months after esophagectomy. *IS* incentive spirometry; *IMT* inspiratory muscle training

Between September 2020 and August 2023, 115 patients with thoracic or abdominal esophageal squamous cell cancer underwent esophagectomy at the Kindai University Hospital. Among the 115 patients, 73 met the exclusion criteria, and 42 were eligible to participate. Eligible participants were randomly assigned to one of the two groups. One patient from each group dropped out during the intervention period. Forty patients completed the intervention and underwent follow-up at T1. Two patients who dropped out refused respiratory training because of the severe side effects of NAC. In the postoperative analysis, three patients in the IS group and four patients in the IMT group could not be followed up at 1 and 3 months after esophagectomy. In addition, one patient in the IMT group was diagnosed with postoperative phrenic nerve palsy (Fig. 1). Three and five patients in the IS and IMT groups, respectively, were unable to be followed up for both T2 and T3, of whom one patient each in the IS and IMT groups was unable to follow up owing to early postoperative metastasis or recurrence during treatment. Additionally, one and three patients in the IS and IMT groups, respectively, declined to be followed up (evaluation and measurement) owing to fatigue and anorexia. Finally, one patient in the IS group required prolonged hospitalization owing to severe infection-related suture failure, and one patient in the IMT group could not be followed up owing to phrenic nerve palsy, which resulted in fatigue and dyspnea. Patients who could be followed at T2 or T3 were included in the primary analysis of this study. Of these patients, only one patient in the IMT group was unable to be followed up at T2. The patient in the IMT group who was unable to be followed at T2 declined to undergo evaluation and measurement owing to fatigue and anorexia. Moreover, three patients and one patient in the IS and IMT groups, respectively, were unable to be followed at T3. Three patients in the IS group who declined to undergo evaluation and measurement at T3 were unable to be followed up owing to early postoperative metastasis or recurrence. One patient in the IMT group who declined to undergo evaluation and measurement at T3 was unable to be followed up because of fatigue and anorexia. No harmful events corresponding to the CONSORT Harms 2022 statement were observed. The median number of weeks in training was 7.7 ± 2.0 and 7.4 ± 1.5 weeks in the IS and IMT groups, respectively. The achievement rates of training within the intervention period in the IS and IMT groups were 81.8 ± 6.2% and 80.7 ± 6.6%, respectively (*p* = 0.63).

Table 1 shows that no significant differences were observed in patient characteristics between the IS and IMT groups at baseline. Regarding thoracic surgery procedures, three patients underwent thoracotomy (IS group, *n* = 2; IMT group, *n* = 1). In contrast, 29 patients underwent thoracoscopy (IS group, *n* = 15; IMT group, *n* = 14). Regarding abdominal surgical modalities, 31 of 32 overall included patients underwent hand-assisted laparoscopic surgery (HALS), and one patient in the IS group underwent an open abdominal operation. All of the included patients underwent reconstruction with a gastric tube, and 18 and 14 patients underwent lymphadenectomy in two and three fields, respectively. Furthermore, there were no significant differences between the two groups in surgical data, operative procedures, operation time, and amount of blood loss in esophagectomy procedures. The four patients who developed PPCs were all in the IS group and all were Clavien–Dindo classification grade II [22]. Two patients had pneumonia and two had atelectasis, and only those patients were treated medically with antibiotics in any of those cases. None of the patients had difficulty with self-sputum expectoration, and no bronchoscopy was used to perform sputum suctioning. Two patients in the IS group and one patient in the IMT group underwent cricothyrotomy (mini-tracheostomy) to secure and prevent airway narrowing due to vocal cord edema. No patient in either group was treated with initial ventilatory support for more than 48 h and reintubation due to respiratory failure. Three (17.7%) and four (26.7%) patients in the IS and IMT groups, respectively, had other surgery-related complications. In the IS group, two patients had suture failure owing to surgical site infection and one patient had recurrent nerve palsy. In the IMT group, one patient had chylothorax and three patients had recurrent nerve palsy.

**Table 1:** Comparison of patient backgrounds between the two groups at baseline

	All patients *n* = 32	IS group *n* = 17	IMT group *n* = 15	*p* value
Age (years)	69.2 ± 6.8	68.5 ± 8.7	69.9 ± 4.3	0.56
Male, *n* (%)	22 (68.8)	13 (76.5)	9 (60.0)	0.54
Clinical stage I/II/III/IV	1/9/19/3	0/4/12/1	1/5/7/2	0.58
Body Mass Index	21.2 ± 2.5	21.7 ± 2.4	20.7 ± 2.8	0.27
ASMI (kg/m^2^)	7.0 ± 1.2	7.2 ± 1.0	6.7 ± 1.5	0.32
Hand grip (kg)	32.5 ± 9.0	33.9 ± 8.3	31.0 ± 10.1	0.38
History smoking, *n* (%)	23 (71.9)	13 (76.5)	10 (66.7)	0.83
Peak VO_2_ (ml/kg/min)	18.4 ± 4.8	17.6 ± 5.0	19.3 ± 4.8	0.34
6MWD (m)	501.3 ± 60.7	485.5 ± 57.1	519.3 ± 63.6	0.12
Pulmonary function test				
% pred. FVC (%)	97.8 ± 14.1	96.1 ± 12.2	99.8 ± 16.7	0.48
% pred. FEV_1_ (%)	90.4 ± 14.4	88.5 ± 14.6	92.5 ± 14.9	0.45
FEV_1_/FVC (%)	75.9 ± 9.3	76.8 ± 9.4	74.9 ± 9.8	0.59
IC (L)	2.2 ± 0.5	2.2 ± 0.5	2.1 ± 0.6	0.67
MIP (cmH_2_O)	69.4 ± 25.5	69.4 ± 24.7	69.4 ± 28.0	> 0.99
% pred. MIP (%)	100.9 ± 27.8	98.3 ± 30.9	103.9 ± 25.7	0.58
DE (mm)	56.9 ± 12.0	59.2 ± 11.0	54.4 ± 13.3	0.27
CCI	0.4 ± 0.7	0.5 ± 0.6	0.3 ± 0.8	0.43
COPD, *n* (%)	1 (3.1)	1 (5.9)	0 (0.0)	> 0.99
Diabetes, *n* (%)	6 (18.8)	3 (17.7)	3 (20.0)	> 0.99
NAC regimen				
DCF, *n* (%)	26 (81.3)	13 (76.5)	13 (86.7)	0.78
UDON, *n* (%)	6 (18.8)	4 (23.5)	2 (13.3)	0.78
Surgical data				
Thoracotomy, *n* (%)	3 (9.4)	2 (11.8)	1 (6.7)	> 0.99
Thoracoscopy, *n* (%)	29 (90.6)	15 (88.2)	14 (93.3)	> 0.99
HALS, *n* (%)	31 (96.9)	16 (94.1)	15 (100.0)	0.84
Lymph nodes dissected in 2 fields/3 fields, *n* (%)	18 (56.3)/14 (43.8)	10 (58.8)/7 (41.2)	8 (53.3)/7 (46.7)	> 0.99
Duration of surgery (min)	641.1 ± 107.9	641.1 ± 102.3	641.1 ± 121.0	> 0.99
Blood loss (ml)	369.6 ± 271.1	446.1 ± 327.6	282.9 ± 173.8	0.10
PPCs Grade II, *n* (%)	4 (12.5)	4 (23.5)	0 (0.0)	0.14
Pneumonia, *n* (%)	2 (6.3)	2 (11.8)	0 (0.0)	0.52
Atelectasis, *n* (%)	2 (6.3)	2 (11.8)	0 (0.0)	0.52
Other surgery-related complications, *n* (%)	7 (21.9)	3 (17.7)	4 (26.7)	0.68
Recurrent nerve paralysis, *n* (%)	4 (12.5)	1 (5.9)	3 (20.0)	0.32
Chylothorax, *n* (%)	1 (3.1)	0 (0.0)	1 (6.7)	0.46
Surgical site infection, *n* (%)	2 (6.3)	2 (11.8)	0 (0.0)	0.48
LOS, (days)	28.2 ± 10.4	26.9 ± 6.6	29.7 ± 13.8	0.45

All data are presented as mean ± standard deviation

*ASMI* appendicular skeletal muscle index; *CCI* Charlson comorbidity index; *COPD* chronic obstructive pulmonary disease; *DCF* docetaxel, cisplatin, and 5-fluorouracil; *DE* diaphragmatic excursion; *FEV*_*1*_ forced expiratory volume in a second; *FVC* forced vital capacity; *HALS* hand-assisted laparoscopic surgery; *IC* inspiratory capacity; *IS* incentive spirometry; *IMT* inspiratory muscle training; *m* meters; *MIP* maximal inspiratory pressure; *NAC* neoadjuvant chemotherapy; *peak VO*_*2*_ peak oxygen consumption; *PPCs* postoperative pulmonary complications; *%pred* percent predicted; *LOS* length of hospital stay; *UDON* 5-fluorouracil, docetaxel, and nedaplatin; *VC* vital capacity; *6MWD* 6 min walking distance (in meters)

*p < 0.05, comparison between the two groups

### Change from baseline between both groups

**Fig. 2:**
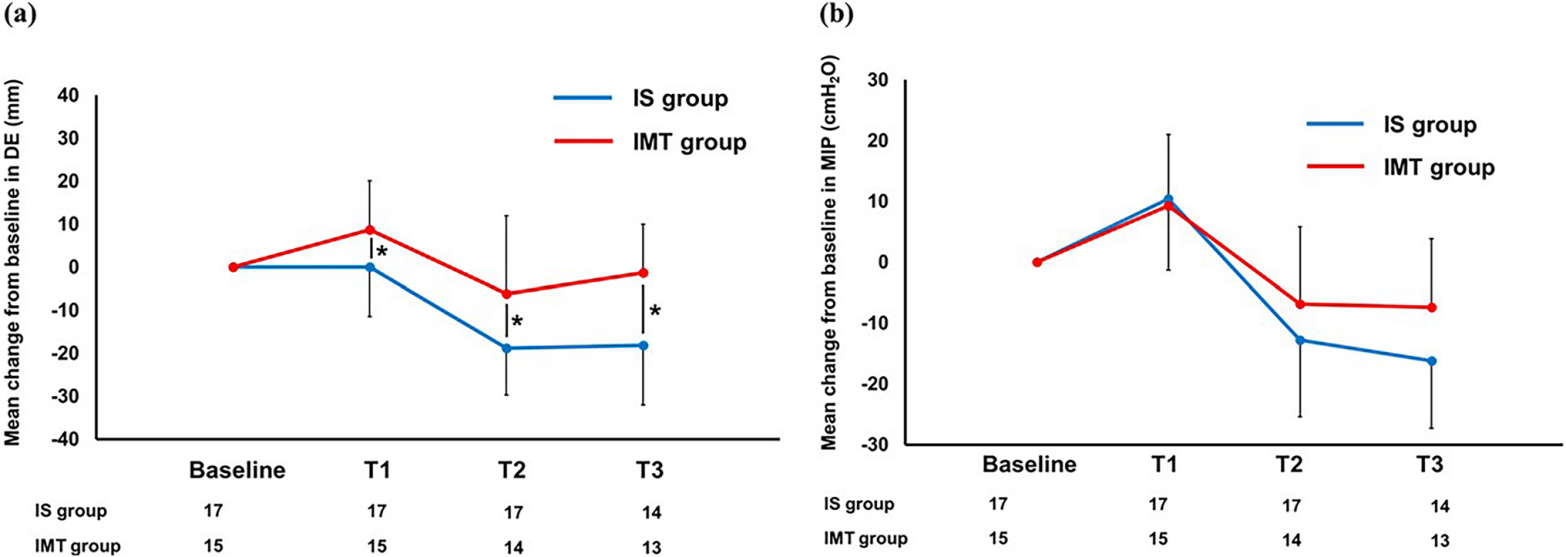
Mean change from baseline to post-operation T3 of the **a** DE and **b** MIP. Baseline: day before initiation of neoadjuvant therapy. T1: few days before esophagectomy. T2: 1 month after esophagectomy. T3: 3 months after esophagectomy. *p < 0.05, comparison between the groups. *DE* diaphragmatic excursion; *IS* incentive spirometry; *IMT* inspiratory muscle training; *MIP* maximal inspiratory pressure

Table 2 shows the comparison of the preoperative and postoperative mean changes from baseline for each measurement. The extent of the mean change in the DE from baseline to preoperative T1 was significantly greater in the IMT group than in the IS group (*p* < 0.05). Although the DE decreased from preoperative T1 to postoperative T2 and T3 in both groups, the mean change in the DE after esophagectomy was significantly smaller in the IMT group than in the IS group (*p* < 0.05; Fig. 2a). The MIP in both groups significantly increased from baseline to preoperative T1 (*p* < 0.05; Online Resource 3). Although the MIP post-op T2 in the IS and IMT groups were lower than that at baseline, only the IS group had a significantly lower MIP at T2 than at baseline (Online Resource 3). Furthermore, the MIP at T3 in the IMT group recovered to the baseline value (Online Resource 3). The mean change in MIP from baseline to postoperative T2 and T3 tended to be smaller in the IMT group than in the IS group; however, there was no significant difference between the two groups (Fig. 2b). The mean change from baseline to post-op T2 and T3 in the pulmonary function and exercise tolerance parameters was not significantly different between the two groups.

**Table 2:** Comparison of the mean change from baseline in each parameter between the two groups

Mean change from baseline	IS group (*n* = 17)	IMT group (*n* = 15)	*p* value
*DE (mm)*			
T1	0.0 ± 11.8	8.8 ± 11.3	0.04*
T2	– 18.8 ± 11.3	– 6.2 ± 18.1	0.02*
T3	– 18.1 ± 14.5	– 1.3 ± 11.2	< 0.01*
*MIP (cmH*_*2*_*O)*			
T1	10.4 ± 11.3	9.3 ± 11.7	0.79
T2	– 12.8 ± 12.9	– 6.8 ± 12.6	0.21
T3	– 16.2 ± 28.4	– 7.3 ± 11.2	0.31
*% pred. MIP (%)*			
T1	15.3 ± 14.1	16.7 ± 20.1	0.81
T2	1.0 ± 24.2	3.2 ± 34.5	0.83
T3	4.2 ± 28.9	10.1 ± 26.5	0.60
*% pred. FVC (%)*			
T1	1.5 ± 5.8	– 0.8 ± 4.6	0.24
T2	– 13.5 ± 12.7	– 14.1 ± 5.3	0.87
T3	– 8.5 ± 10.2	– 10.6 ± 11.0	0.64
*% pred. FEV*_*1*_* (%)*			
T1	3.0 ± 9.1	– 2.3 ± 5.4	0.06
T2	– 7.5 ± 7.7	– 11.3 ± 7.1	0.18
T3	– 2.3 ± 9.8	– 3.8 ± 10.2	0.71
*Peak VO*_*2*_* (ml/kg/min)*			
T1	– 1.1 ± 2.6	– 1.8 ± 1.8	0.36
T2	– 4.2 ± 3.9	– 5.0 ± 2.6	0.51
T3	– 3.4 ± 7.0	– 3.0 ± 3.3	0.86
*6MWD (m)*			
T1	15.5 ± 35.4	8.7 ± 50.7	0.66
T2	– 33.0 ± 43.8	– 59.7 ± 55.1	0.14
T3	1.7 ± 26.1	– 24.8 ± 41.3	0.07

All data were presented as mean ± standard deviation

*DE* diaphragmatic excursion; *FEV*_*1*_ forced expiratory volume in a second; *FVC* forced vital capacity; *IS* incentive spirometry; *IMT* inspiratory muscle training; *m* meters; *MIP* maximal inspiratory pressure; *peak VO*_*2*_ peak oxygen consumption; *6MWD* 6 min walking distance (in meters)

*p < 0.05, comparison between the two groups for the mean change from baseline

## Discussion

In this study, we examined whether preoperative IMT could inhibit postoperative decline in the DE and MIP. The results showed that the decline in postoperative DE was significantly inhibited in the IMT group compared to the IS group, and postoperative MIP tended to be relatively preserved in the IMT group. Our study is the first to report the diaphragmatic function-enhancing effect of preoperative IMT in patients with esophageal cancer, which is preserved for at least 3 months postoperatively. In a previous study, we reported the superiority of preoperative IMT in enhancing the DE and preventing PPCs. The MIP increased significantly during the preoperative period in both the IS and IMT groups; however, the DE significantly increased only in the IMT group [8].

Randomized-controlled trials investigating IMT in healthy participants and patients with stroke reported an increase in diaphragm muscle thickness after approximately 6–8 weeks of intervention [23, 24]. An increase in type I, IIa, and IIb fibers in the diaphragm was observed in rats after 8 weeks of low-to-moderate inspiratory resistance [25]. The average duration of IMT in our study was sufficient to improve the DE before esophagectomy; however, muscle degeneration in the diaphragm, decreased muscle output, and decreased mobility were observed after thoracic and abdominal surgery. Welvaart et al. found that the reduction in muscle force and degeneration of the myosin heavy chain were observed in type II fibers of the diaphragm at 2 h after thoracic surgery [26]. There are reports stating that the DE was still declining to about 70% of its preoperative value on the seventh postoperative day [27, 28]. In a randomized-controlled trial of preoperative IMT in upper abdominal surgery, the group with preoperative IMT intervention tended to have preserved DE and MIP compared with the control group at 1 month postoperatively [10, 29]. Although these studies support our findings, we followed the effects of preoperative IMT on diaphragmatic function and inspiratory muscle strength over 3 months after esophagectomy. To the best of our knowledge, no other study has investigated whether the effects of preoperative IMT on inspiratory muscles in upper abdominal surgery are maintained for several months postoperatively; hence, this study is the first to report that the DE and MIP is better preserved in the IMT group than in the IS group until 3 months after esophagectomy, despite the thoracic invasion caused by esophagectomy. It can be inferred that preoperative IMT contributes to the postoperative recovery of diaphragmatic function after upper abdominal surgery.

Our results showed that pulmonary function and exercise tolerance in the IMT group were affected by surgical invasion, regardless of preserved postoperative DE and MIP after esophagectomy in the IMT group. A randomized-controlled trial on preoperative IMT for upper abdominal surgery reported that the DE increased after the intervention and was maintained in the IMT group postoperatively, although the pre- and postoperative changes in pulmonary function were not significantly different between the control group [29]. This study showed the same trend as our results. Considering this, we hypothesized that although IMT increased the DE in this study, it did not have a significant effect on pulmonary function, and postoperative pulmonary function was more affected by pain and a limited range of chest movement than by respiratory muscle involvement. In our previous randomized-controlled trial of patients with stable chronic obstructive pulmonary disease (COPD), the DE was enhanced and peak VO_2_ increased with improvement in ventilatory parameters during exercise after 12 weeks of IMT at 50% MIP [30]. On the other hand, an intervention study of IMT with 50% MIP for 4 weeks in healthy participants increased diaphragm muscle thickness and MIP with no change in the peak VO_2_ [31]. The effect of IMT on exercise tolerance is beneficial for diseases that have restricted ventilation during exercise, such as COPD, and is less effective for patients without limited ventilation during exercise. Thus, DE enhancement following preoperative IMT in patients with esophageal cancer may have no significant effect on pulmonary function or exercise tolerance in the preoperative and postoperative periods. As for postoperative clinical outcomes, we cannot state anything with statistical differences in this study, because the sample size is too small, but a large multicenter study in the future may be able to discuss mid- to long-term postoperative clinical outcomes.

Our study had several limitations. First, the sample size was calculated from baseline to the preoperative T1 inspiratory training intervention study, which resulted in a smaller sample size in the postoperative follow-up study. Second, several patients were unable to undergo postoperative DE and MIP measurements at T2 and T3 follow-ups. Furthermore, this study was designed to follow up with patients up to 3 months postoperatively and not beyond that time. Third, our study could not be blinded to the patients, because we performed inspiratory training using dedicated equipment in a randomized-controlled trial. Finally, although we have recorded the trend of MIP over time every 2 weeks during the preoperative IS/IMT intervention period, but no data on DE during the preoperative IS/IMT intervention period.

## Conclusion

Preoperative IMT in patients with thoracic and abdominal esophageal cancers enhanced diaphragmatic function, which was preserved over the 3-month postoperative period. Inspiratory muscle strength was increased by preoperative IMT and tended to be preserved for at least 3 months after esophagectomy. However, pulmonary function and exercise tolerance were more strongly affected by surgical invasion and were not compensated by the preserved diaphragmatic function.

## Supplementary Information

Below is the link to the electronic supplementary material.

Supplementary file1 (DOCX 486 KB)

## Data Availability

All data generated or analyzed during this study are included in this article and its online supplementary material. Further inquiries can be directed to the corresponding authors.
