# Supplementary material for: Preoperative inspiratory muscle training preserved diaphragmatic excursion after esophagectomy: a randomized-controlled trial
Source: Esophagus. 2025 Apr 3;22(3):331–9. doi: 10.1007/s10388-025-01123-w (PMC12167333; doi:10.1007/s10388-025-01123-w)
Supplement: Supplementary file 1 — Supplementary file1 (DOCX 486 KB) [file 10388_2025_1123_MOESM1_ESM.docx]

**Supplementary Materials**

**Preoperative inspiratory muscle training preserved diaphragmatic excursion after esophagectomy: A randomized controlled trial**


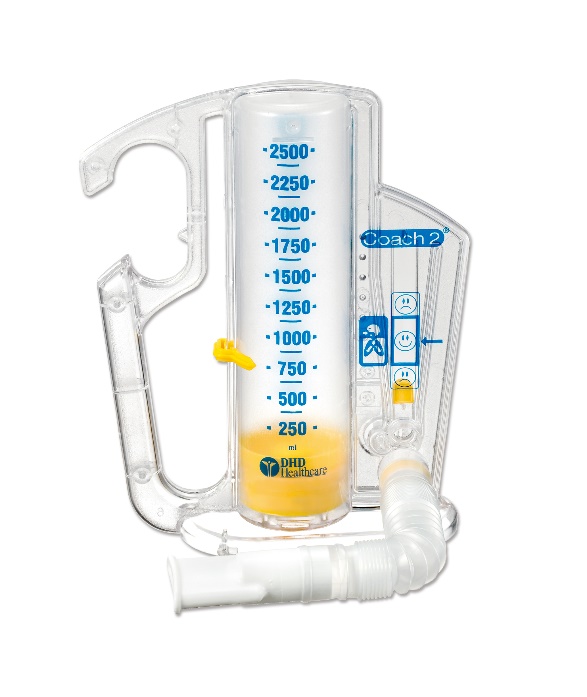

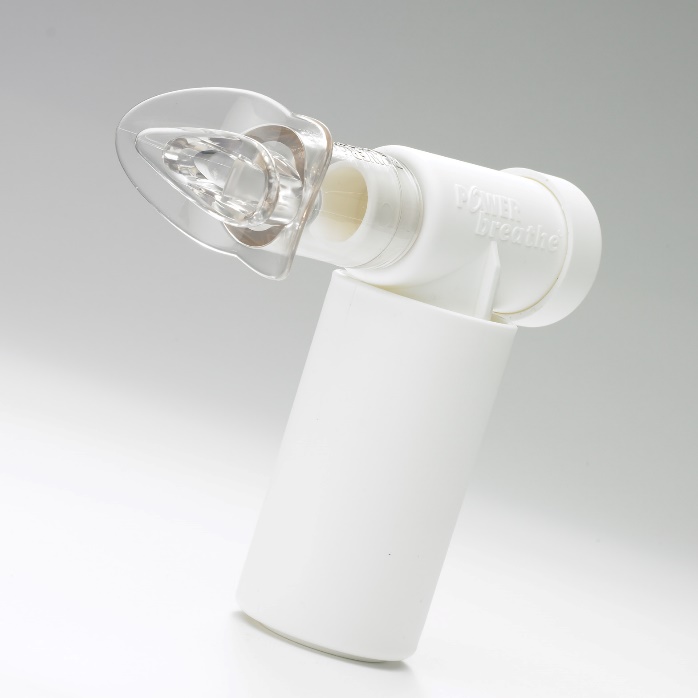


**(a)**

**(b)**

Supplementary Figure 1

(a) Image of Coach2^®^Incentive Spirometer 2500 ml, which was used in the incentive spirometry group.

(b) Image of POWERbreath^®^ medic, which was used in the inspiratory muscle training group.


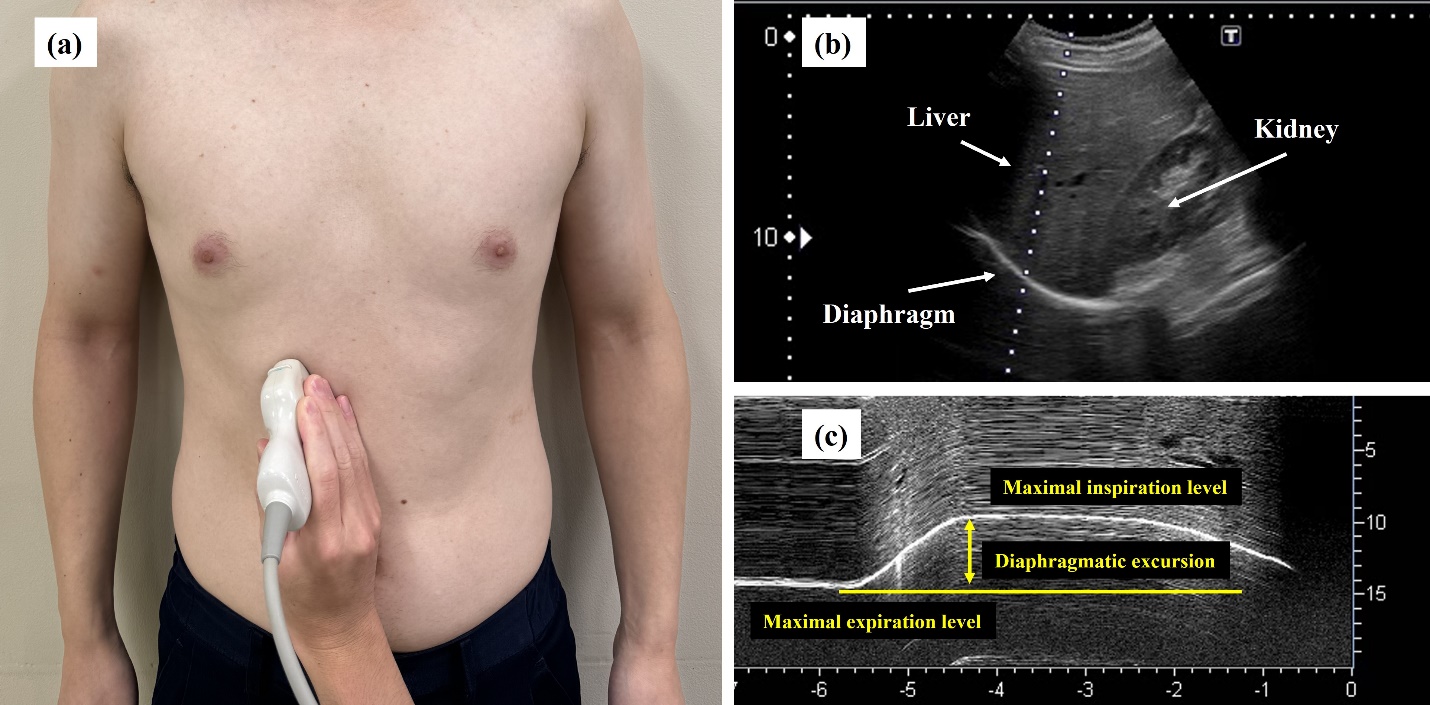


Supplementary Figure 2

(a) The right diaphragm was identified by placing a convex probe under the right costal arch along the rib margin. This measurement was conducted in the standing position.

(b) Two-dimensional ultrasonographic image of the right hemidiaphragm (B-mode).

(c) Diaphragmatic displacement (diaphragmatic excursion) was measured between maximal expiration level and maximal inspiration level (M-mode).


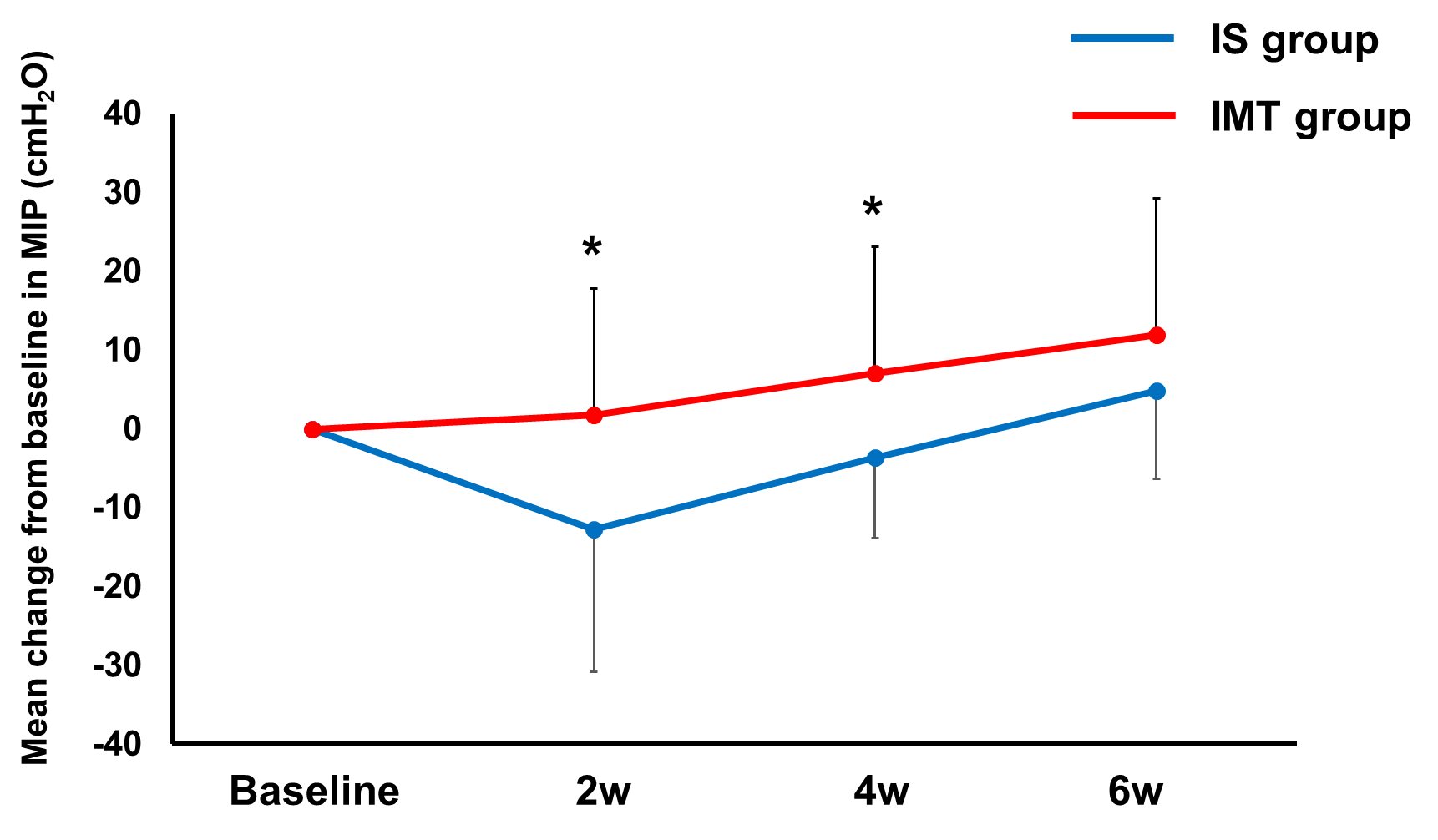


Supplementary Figure 3. Mean change from baseline to pre-operation of the MIP

Baseline, the day prior to NAC initiation; 2w, 2 weeks from initiation of respiratory training; 4w, 4 weeks from initiation of respiratory training; 6w, 6 weeks from initiation of respiratory training

**p*<0.05, comparison between the groups

DE, diaphragmatic excursion; IS, incentive spirometry; IMT, inspiratory muscle training; NAC, neoadjuvant chemotherapy; MIP, maximal inspiratory pressure

Supplementary Table 1.

Paired-test results at each time point were compared to baseline T0 within each group.

|  | IS group (n=17) | | | |  |  | IMT group (n=15) | | | |  |
| --- | --- | --- | --- | --- | --- | --- | --- | --- | --- | --- | --- |
|  | Baseline | T1 | T2 | T3 | *p* value* |  | Baseline | T1 | T2 | T3 | *p* value* |
| MIP, cmH_2_O | 69.4±23.9 | 79.8±26.0 ^†^ | 56.6±19.2 ^†, ‡^ | 53.9±19.7 ^‡^ | <.01* |  | 69.4±27.1 | 78.7±23.1 ^†^ | 57.0±22.5 ^‡^ | 64.8±24.5 ^‡^ | <.01* |
| % pred. MIP, % | 98.3±30.9 | 113.5±31.9 ^†^ | 99.2±31.8 ^‡^ | 97.2±38.0 | .04* |  | 103.9  ± 25.7 | 120.6±25.5 | 101.8±34.9 | 108.9±28.4 | .36 |
| DE, mm | 59.2±11.0 | 59.2±10.7 | 40.4±12.0 ^†, ‡^ | 44.8±6.6 ^†, ‡^ | <.01* |  | 54.4±13.3 | 63.2±13.9 | 47.2±17.9 ^‡^ | 54.0±14.6 | <.01* |
| % pred. FVC, % | 96.1±12.2 | 97.6±12.3 | 83.6±13.1 ^†, ‡^ | 90.4±10.4 ^‡^ | <.01* |  | 99.8±16.7 | 99.0±15.7 | 85.6±16.8 ^†, ‡^ | 88.0±19.8 ^†, ‡^ | <.01* |
| % pred. FEV_1_, % | 88.5±14.6 | 91.5±16.4 | 82.4±13.0 ^†, ‡^ | 86.8±11.0 ^§^ | .03* |  | 92.5±14.9 | 90.2±14.9 | 81.2±13.2 ^†, ‡^ | 86.8±17.5 | <.01* |
| IC, L | 2.2±0.5 | 2.3±0.5 | 1.7±0.4 ^†, ‡^ | 1.8 ±0.4 ^†, ‡^ | <.01* |  | 2.1±0.6 | 2.2±0.6 | 1.9±0.6 ^†^ | 1.8±0.7 ^†, ‡^ | <.01* |
| Peak VO_2_, ml/kg/min | 17.6±5.0 | 16.6±4.3 | 13.4±3.7 ^†, ‡^ | 15.7±4.2 | .03* |  | 19.3±4.8 | 17.5±4.0 ^†^ | 13.9±3.1 ^†, ‡^ | 16.2±3.3 ^†^ | <.01* |
| 6MWD, m | 485.5±57.1 | 501.0±58.7 | 452.5±59.4 ^†, ‡^ | 485.9 ±61.6 ^§^ | <.01* |  | 519.3 ±63.6 | 528.0 ±48.7 | 454.2 ±57.3 ^†, ‡^ | 497.4 ±60.6 ^§^ | <.01* |

All data were presented by [mean±SD]

**p*<0.05, Comparison across the four groups, ^†^*p*<0.05 comparison vs. timepoint 0 (Baseline), ^‡^*p*<0.05 Comparison vs. T1 (pre-operation), ^§^*p*<0.05 Comparison vs. T2 (post-operation at 1 month).

DE, diaphragmatic excursion; FEV_1_, forced expiratory volume in a second; FVC, forced vital capacity; IC, inspiratory capacity; IS, incentive spirometry; IMT, inspiratory muscle training; MIP, maximal inspiratory pressure; peak VO_2_, peak oxygen consumption; 6MWD, 6-minutes walking distance.

**Supplementary Table 2**. Comparison of mean change from baseline for each parameter in patients with thoracoscopy procedure between the two groups

| **Mean change from baseline** | **IS group (n=15)** | **IMT group (n=14)** | ***p*-value** |
| --- | --- | --- | --- |
| DE (mm) |  |  |  |
| T1 | -0.2±10.5 | 8.8±11.7 | 0.04^*^ |
| T2 | -18.5±10.6 | -5.3±18.8 | 0.03^*^ |
| T3 | -16.0±14.4 | 0.2±11.6 | <0.01^*^ |
| MIP (cmH_2_O) |  |  |  |
| T1 | 10.8±11.4 | 8.1±11.1 | 0.51 |
| T2 | -14.0±12.4 | -7.1±13.1 | 0.16 |
| T3 | -16.4±29.5 | -7.5±11.7 | 0.34 |
| % pred. MIP (%) |  |  |  |
| T1 | 15.6±13.9 | 14.2±18.2 | 0.82 |
| T2 | -0.9±24.5 | 4.0±35.8 | 0.67 |
| T3 | 3.7±31.2 | 11.9±27.0 | 0.51 |
| % pred. FVC (%) |  |  |  |
| T1 | 1.6±5.8 | -1.4±4.0 | 0.12 |
| T2 | -14.3±13.3 | -14.8±4.9 | 0.90 |
| T3 | -8.1±10.6 | -11.8±10.8 | 0.45 |
| % pred. FEV_1_ (%) |  |  |  |
| T1 | 3.1±9.4 | -3.1±4.4 | 0.03^*^ |
| T2 | -8.1±7.1 | -11.8±7.2 | 0.20 |
| T3 | -2.4±9.0 | -4.4±10.4 | 0.64 |
| Peak VO_2_ (mL/kg/min) |  |  |  |
| T1 | -1.3±2.7 | -2.1±1.5 | 0.33 |
| T2 | -4.5±4.0 | -5.4±2.3 | 0.47 |
| T3 | -3.8±7.6 | -3.5±3.1 | 0.90 |
| 6MWD (m) |  |  |  |
| T1 | 16.4±37.0 | 0.1±39.6 | 0.26 |
| T2 | -32.8±45.3 | -62.5±56.4 | 0.14 |
| T3 | 6.4±25.6 | -24.8±41.3 | 0.04^*^ |
|  |  |  |  |
| PPCs Grade Ⅱ, n (%) | 2 (13.3) | 0 (0.0) | 0.48 |

All data are presented as mean±standard deviation

^*^*p*<0.05, comparison between the two groups for the mean change from baseline

DE, diaphragmatic excursion; FEV_1_, forced expiratory volume in a second; FVC, forced vital capacity; IS, incentive spirometry; IMT, inspiratory muscle training; m, meters; MIP, maximal inspiratory pressure; peak VO_2_, peak oxygen consumption; PPCs, post-operative pulmonary complications; 6MWD, 6 min walking distance

**Supplementary Table 3**. Comparison of mean change from baseline for each parameter in patients who underwent HALS between the two groups

| **Mean change from baseline** | **IS group (n=16)** | **IMT group (n=15)** | ***p-*value** |
| --- | --- | --- | --- |
| DE (mm) |  |  |  |
| T1 | - 0.2±12.2 | 8.8±11.3 | 0.04^*^ |
| T2 | -18.3±11.5 | -5.2±18.1 | 0.02^*^ |
| T3 | -14.5±11.0 | -0.3±11.2 | <0.01^*^ |
| MIP (cmH_2_O) |  |  |  |
| T1 | 9.8±11.4 | 9.3±11.7 | 0.91 |
| T2 | -13.0±13.3 | -6.8±12.6 | 0.20 |
| T3 | -9.7±15.3 | -7.3±11.2 | 0.66 |
|  |  |  |  |
| PPCs Grade Ⅱ, n (%) | 3 (18.8%) | 0 (0.0%) | 0.23 |

All data were presented as mean±standard deviation

^*^*p*<0.05, comparison between the two groups for the mean change from baseline

DE, diaphragmatic excursion; HALS, hand-assisted laparoscopic surgery; IS, incentive spirometry; IMT, inspiratory muscle training; MIP, maximal inspiratory pressure; PPCs, post-operative pulmonary complications

**Supplementary Table 4**. Comparison of baseline background in ITT analysis between the two groups and the amount of change in the main results before and after the intervention.

|  | **All patients**  **n=40** | **IS group**  **n=20** | **IMT group**  **n=20** | ***p*-value** |
| --- | --- | --- | --- | --- |
| Age (years) | 68.4±6.7 | 67.8±8.4 | 69.0±4.8 | 0.58 |
| Male, n (%) | 28 (70.0) | 14 (70.0) | 14 (70.0) | >0.99 |
| Clinical stage Ⅰ/Ⅱ/Ⅲ/Ⅳ | 0/10/24/5 | 0/5/14/1 | 0/6/10/4 | 0.35 |
| Body mass index | 21.2±2.5 | 21.7±2.4 | 20.6±2.5 | 0.18 |
| ASMI (kg/m^2^) | 7.0±1.1 | 7.0±1.0 | 6.9±1.3 | 0.67 |
| Hand grip test (kg) | 32.5±8.9 | 32.7±8.7 | 32.3±9.5 | 0.90 |
| History of smoking, n (%) | 31 (77.5) | 16 (80.0) | 15 (75.0) | >0.99 |
| Peak VO_2_ (mL/kg/min) | 18.3±4.8 | 17.2±5.0 | 19.4±4.5 | 0.16 |
| 6MWD (m) | 493.4±60.8 | 482.1±59.1 | 504.7±63.5 | 0.25 |
| Pulmonary function test |  |  |  |  |
| % pred. FVC (%) | 97.4±14.2 | 97.0±12.3 | 97.8±16.5 | 0.86 |
| % pred. FEV_1_ (%) | 89.8±14.3 | 88.9±15.0 | 90.7±14.4 | 0.71 |
| FEV_1_/FVC (%) | 75.7±9.0 | 76.0±9.0 | 75.4±9.4 | 0.82 |
| IC (L) | 75.7±9.0 | 76.0±9.0 | 75.4±9.4 | 0.82 |
| MIP (cmH_2_O) | 66.9±24.2 | 67.3±23.6 | 66.4±26.0 | 0.91 |
| % pred. MIP (%) | 97.3±28.5 | 97.6±30.4 | 96.9±28.0 | 0.94 |
| DE (mm) | 57.9±12.6 | 59.9±11.7 | 55.7±14.2 | 0.22 |
| CCI [median; IQR] | 0 [0; 1] | 0 [0; 1] | 0 [0; 0.3] | 0.44 |
| COPD, n (%) | 2 (5.0) | 1 (5.0) | 1 (5.0) | >0.99 |
| Diabetes, n (%) | 7 (17.5) | 3 (15.0) | 4 (20.0) | >0.99 |
| NAC regimen |  |  |  |  |
| DCF / UDON, n (%) | 34 (85.0) / 6 (15.0) | 16 (80.0) / 4 (20.0) | 18 (90.0) / 2 (10.0) | 0.66 |
| Surgical data |  |  |  |  |
| Thoracotomy, n (%) | 8 (20.5) | 4 (20.0) | 4 (20.0) | >0.99 |
| Thoracoscope, n (%) | 31 (79.5) | 16 (80.0) | 15 (75.0) | >0.99 |
| HALS, n (%) | 38 (95.0) | 18 (90.0) | 20 (100.0) | 0.49 |
| Lymph nodes dissected in 2 fields / 3 fields, n (%) | 21 (52.5) /  19 (47.5) | 13 (58.8) /  7 (35.0) | 8 (40.0) /  12 (60.0) | 0.21 |
| Duration of surgery (min) | 652.8±119.4 | 640.1±113.9 | 665.6±129.9 | 0.53 |
| Blood loss (ml) | 397.9±284.1 | 445.5±330.1 | 347.8±233.8 | 0.30 |
| PPCs Grade Ⅱ, n (%) | 5 (12.5) | 4 (20.0) | 1 (5.0) | 0.34 |
| Pneumonia, n (%) | 2 (5.0) | 2 (11.8) | 0 (0.0) | 0.49 |
| Atelectasis, n (%) | 3 (7.5) | 2 (11.8) | 1 (5.0) | >0.99 |
| Other surgery-related complications | 14 (35.0) | 5 (25.0) | 9 (45.0) | 0.32 |
| Recurrent nerve paralysis, n (%) | 6 (15.0) | 4 (20.0) | 5 (25.0) | 0.70 |
| Chylothorax, n (%) | 2 (5.0) | 1 (5.0) | 1 (5.0) | >0.99 |
| Surgical site infection, n (%) | 4 (10.0) | 3 (15.0) | 1 (5.0) | 0.61 |
| Phrenic nerve paralysis, n (%) | 1 (2.5) | 0 (0.0) | 1 (5.0) | >0.99 |
| Lymphatic leakage, n (%) | 1 (2.5) | 0 (0.0) | 1 (5.0) | >0.99 |
| LOS, (days) | 36.2±27.2 | 33.4±30.9 | 39.1±24.1 | 0.52 |
|  |  |  |  |  |
| ΔDIE | 4.0±13.5 | −2.3 (13.7) | 10.3 (10.5) | <.01* |
| ΔMIP | 11.6±12.5 | 9.3±10.9 | 13.8±14.0 | 0.27 |
| Δ% pred. MIP (%) | 17.3±17.4 | 0.05±2.52 | 0.14±1.29 | 0.88 |
| Δ% pred. FVC (%) | 0.92±6.2 | 2.9±6.9 | -1.1±5.0 | 0.04 |
| ΔPeak VO_2_ (ml/kg/min) | -1.7±2.3 | −1.3±2.5 | −2.0±2.1 | 0.38 |
| Δ6MWD (m) | 11.0±41.3 | 11.2±34.3 | 10.9±49.1 | >0.99 |

All data were presented as mean±standard deviation

**p*<0.05, comparison between the two groups

ASMI, appendicular skeletal muscle index; CCI, Charlson comorbidity index; COPD; chronic obstructive pulmonary disease; DCF, docetaxel, cisplatin, and 5-fluorouracil; DE, diaphragmatic excursion; FEV1, forced expiratory volume in a second; FVC, forced vital capacity; HALS, hand-assisted laparoscopic surgery; IC, inspiratory capacity; IS, incentive spirometry; IMT, inspiratory muscle training; IQR< interquartile range; MIP, maximal inspiratory pressure; NAC, neoadjuvant chemotherapy; peak VO_2_: peak oxygen consumption; PPCs, postoperative pulmonary complications; %pred, percent predicted; LOS, length of stay; UDON, 5-fluorouracil, docetaxel, and nedaplatin; VC, vital capacity; 6MWD, 6 min walking distance
